# Construction and characterization of an infectious replication competent clone of porcine endogenous retrovirus from Chinese miniature pigs

**DOI:** 10.1186/1743-422X-10-228

**Published:** 2013-07-09

**Authors:** Silong Xiang, Yuyuan Ma, Qipo Yan, Maomin Lv, Xiong Zhao, Huiqiong Yin, Nian Zhang, Junting Jia, Rong Yu, Jingang Zhang

**Affiliations:** 1 Key Laboratory of Drug Targeting and Drug Delivery System, Ministry of Education, West China School of Pharmacy, Sichuan University, Chengdu 610041, China; 2 Laboratory for Viral Safety of National Centre of Biomedical Analysis, Beijing Institute of Transfusion Medicine, Beijing 100850, China

**Keywords:** Chinese miniature pigs, Porcine endogenous retroviruses, Infectious molecular clone, Xenotransplantation

## Abstract

**Background:**

Xenotransplantation from animals has been considered to be a preferable approach to alleviate the shortage of human allografts. Pigs are the most suitable candidate because of the anatomical and physiological similarities shared with humans as well as ethical concerns. However, it may be associated with the risk of transmission of infectious porcine pathogens. Porcine endogenous retroviruses (PERVs) are of particular concern because they have been shown to infect human cells *in vitro.* To date, researches on the molecular characteristics and potential pathogenicity of PERV are still tenuous. In this report, an infectious replication competent clone of PERV from Wuzhishan pigs (WZSPs) in China was generated and characterized. This infectious clone will contribute to studies on PERV virology and control of PERV in xenotransplantation using Chinese miniature pigs.

**Methods:**

The proviral DNA of PERV from WZSPs was amplified in two overlapping halves. Then the two fragments were isolated, subcloned and fused to generate pBluescriptαSK^+^-WZS-PERV recombinant clones. Screened with RT-PCR, a molecular clone of PERV designated as WZS-PERV(2) was selected. Its infectivity and replication competency were characterized in HEK293 cells by PCR, real-time fluorescent quantitative RT-PCR, western blot, indirect immunofluorescence assay as well as sequence analysis.

**Results:**

The ability of WZS-PERV(2) to infect human cells and produce infectious virions were shown after transfection of the clone into HEK293 cells and infection of PERV derived from this recombinant clone. The expression of Gag proteins were detected in HEK293 cells infected with the virus derived from the clone by the indirect immunofluorescence assay and western blot. The results of sequences analysis and comparison combined with the PCR based genotyping result demonstrated that the WZS-PERV(2) belonged to PERV-A subgroup. Compared with a previous proviral DNA clone of PERV (PERV-WZSP), G to A hypermutation occurred in the *env* gene of WZS-PERV(2) was found, whereas APOBEC proteins have the potential to inhibit the replication of a variety of retroviruses through a cDNA cytosine deamination mechanism, so we presumed these G to A hypermutation might be the contribution of porcine APOBEC3F.

**Conclusions:**

Altogether, an infectious replication competent clone of PERV from Chinese miniature pigs (WZSPs) termed WZS-PERV(2) was generated, characterized and sequenced.

## Background

Xenotransplantation from animals has been proposed as a preferable approach to alleviate the shortage of human donor organs. Pigs are the most suitable candidate because of the anatomical and physiological similarities shared with humans as well as ethical concerns [1-3]. However, it may be associated with the risk of transmission of infectious porcine pathogens [4-7]. Most transmissible porcine microorganisms to human recipients can be precluded by specific-pathogen-free (SPF) breeding, but this is not possible in the case of porcine endogenous retroviruseses (PERVs), which are integrated into the genome of all pigs and can stably inherit in the germ line.

PERVs belong to C-type retroviruses and are classified to three subtypes, PERV-A, PERV-B and PERV-C, according to the env sequences [8]. PERV-A and PERV-B are characterized with polytropism and can productively infect a wide spectrum of human and other mammalian cells. In contrast, PERV-C is ecotropic viruses and can only infect pig cell lines. Additionally, PERV A/C recombinants have recently been reported. These recombinants are more infectious to human cells, approximately 500-fold stronger than that of their parental PERV-A [9]. To date, albeit no evidence of PERV infection of xenograft recipients *in vivo* has been found [10-12], it should be noted that PERVs released from PK15 cells, PBMCs and porcine endothelial cells were capable of infecting human cells *in vitro*[13-15]. Therefore, PERV poses a potential risk for clinical xenotransplantation. Furthermore, the possibility to form a chimeric virus when porcine and human retroviruses coinfect a cell is also proclaimed as a potential risk for xenotransplantation.

In view of their clear genetic background, strong disease resistance and tiny interindividual differences, Chinese miniature pigs have been developed as the potential organ donors for xenotransplantation in China [16]. Previously, we performed a large-scale survey on the presence and expression status of PERV in seven breeds of Chinese miniature pigs [17] and the result showed that PERV present in all 348 genomic DNA samples, and no expression of PERV-C was found in Wuzhishan pigs (WZSPs), which were further confirmed to harbor lower PERV copy numbers compared with other breeds [18] and considered to be more suitable for xenotransplantation. Thus it is essential to study the molecular characteristics of PERV from WZSPs.

In this study, we constructed and characterized an infectious replication-competent clone of porcine endogenous retroviruses from Chinese miniature pigs (WZSPs). PERV proviral DNA was amplified in two overlapping halves and the full-length proviral DNAs from WZSPs were cloned based on PCR method. Screening with RT-PCR, we obtained an infectious PERV clone designated as WZS-PERV(2) and its characteristics were analyzed.

## Results and discussion

### Cloning and identification of the full-length PERV provirus

As previously described [19], the proviral DNA of PERV was amplified in two overlapping halves with the genomic DNA of peripheral blood mononuclear cells (PBMCs) from WZSPs as templates. Three pMD18-5’half clones and three pBluescriptαSK^+^-3’ half clones were obtained. Then these two clones were isolated, digested and fused to generate pBluescriptαSK^+^-WZS-PERV recombinant clones. Based on the results of colony PCR and restriction enzyme digestions (data not shown), nine pBluescriptαSK+−WZS-PERV positive clones were created, and designated as WZS-PERV(1–9) respectively so that the infectious molecular clones could be selected and characterized.

### Screening of infectious clones with RT-PCR and subtype analysis

**Figure 1:**
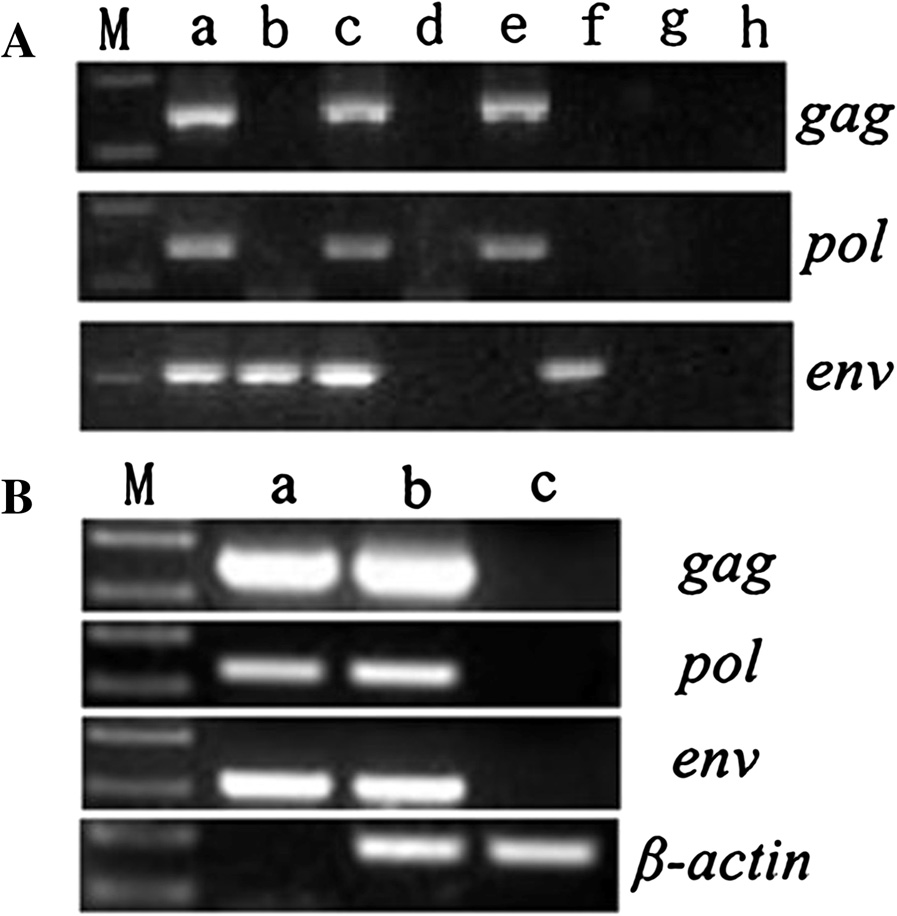
**RT-PCR and PCR analysis of RNA or DNA from the WZS-PERV(2) transfected HEK293 cell or the HEK293 cells infected with PERV derived from WZS-PERV(2). A**. Screening of infectious clones with RT-PCR: *Lane M* DL 2000 DNA Marker; *lane a* PCR products of genomic DNA from PK15 cells; *lane g* PCR products of genomic DNA from fresh HEK293 cells; *lane h* blank control; *lane b-f* RT-PCR products of RNA from the HEK293 cells transfected with 1–5 recombinant plasmids. **B**. PCR products of genomic DNA from the infected cells at 72 hours after infection: *lane a* PCR products of genomic DNA from PK15 cells; *lane b* PCR products of genomic DNA from the infected HEK293 cells; *lane c* PCR products of genomic DNA from fresh HEK293 cells.

To obtain infectious clones, all the 9 reconstructed plasmids were transfected into HEK293 cells with lipofectamine respectively and screened with RT-PCR at 72 hours post-transfection. As revealed by the RT-PCR, only c lane, namely, WZS-PERV(2) clone, present specific bands of all the three genes (Figure 1A, only 1-5 recombinant plasmids were shown), thus indicated that WZS-PERV(2) clone was infectious and replication competent and the PERV-*env* subtype analysis suggested that the WZS-PERV(2) clone belonged to PERV-A (data not shown). Then the WZS-PERV(2) was employed for subsequent molecular characterization.

### Characterization of WZS-PERV(2) clone

#### PCR and real-time fluorescent quantitative PCR

The supernatant of HEK293 cells transfected with WZS-PERV(2) was collected and used to infect fresh HEK293 cells. At 72 hours post-infection, the genomic DNA of the infected cells, fresh HEK293 cells and PK15 cells were isolated and PCR was performed with primers specific for *gag, pol, env* and human *β-actin* genes. The PERV PCR products were detected in the infected HEK293 cells (Figure 1B), suggesting that WZS-PERV(2) was successfully integrated into the genomic DNA of HEK293 cells.

**Figure 2:**
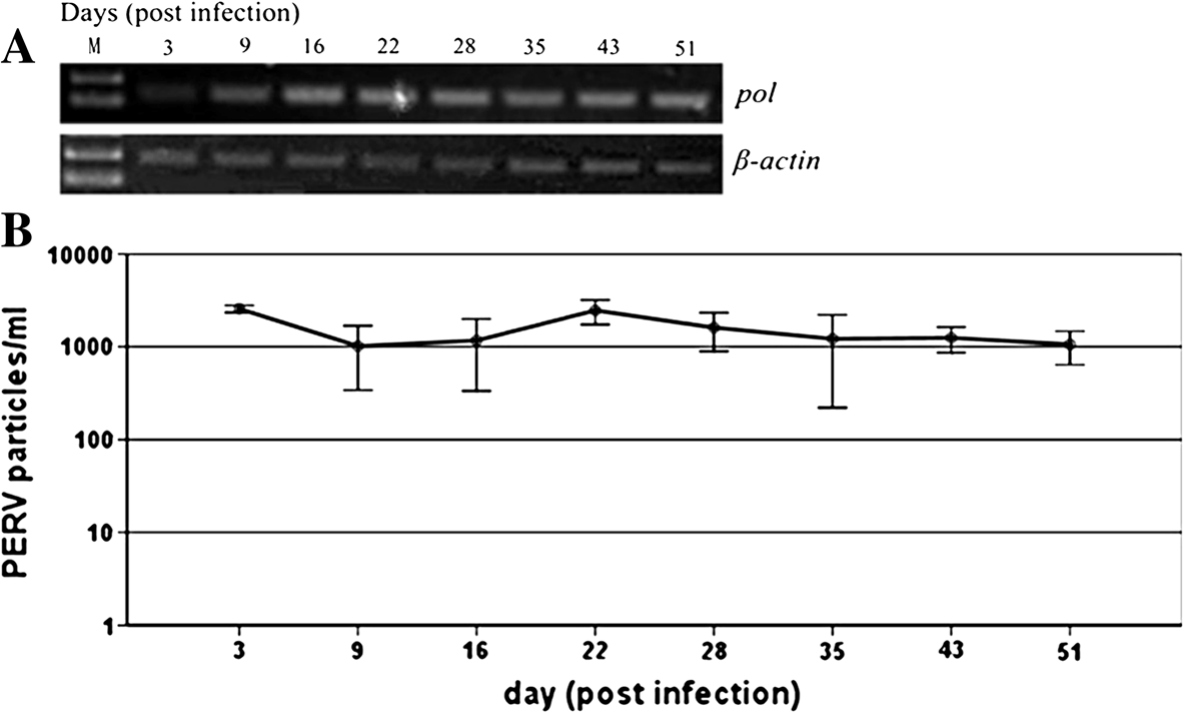
**PCR analysis of cellular DNA from HEK293 cells infected with PERV derived from WZS-PERV(2), and replication kinetics of PERV derived from the clone. A**. PCR products of genomic DNA from HEK293 cells infected with PERV derived from the WZS-PERV(2) clone using the pol and human β-actin genes for 51 days. **B**. Numbers of PERV particles from the supernatants (per ml) during 51 days.

After confirming the presence of PERV derived from WZS-PERV(2) in the infected cells, then these infected cells were serially passaged. The genomic DNA of the infected cells was extracted at every passage time and PCR was employed with the PERV-*pol* specific primer pairs. As revealed by the PERV PCR products, the PERV derived from the WZS-PERV(2) was stably integrated into the genomic DNA of HEK293 cells (Figure 2A).

In the parallel, the cell-free supernatants of infected cells were collected at every passage time, the PERV copies in the supernatants were determined by absolute real time PCR based on SYBR Green I for amplifying *pol* gene and the replication kinetics of PERV derived from WZS-PERV(2) was delineated using SPSS 15.0 software package (Figure 2B). As shown in the replication kinetics, the PERV copy numbers in the supernatants were maintained between 1000 and 2500 particles per ml for 51 days and stable release of PERV virions post infection were observed. Long-term maintenance of the infected cells showed the virus derived from the clone had stable replication competency.

### Western blot

**Figure 3:**
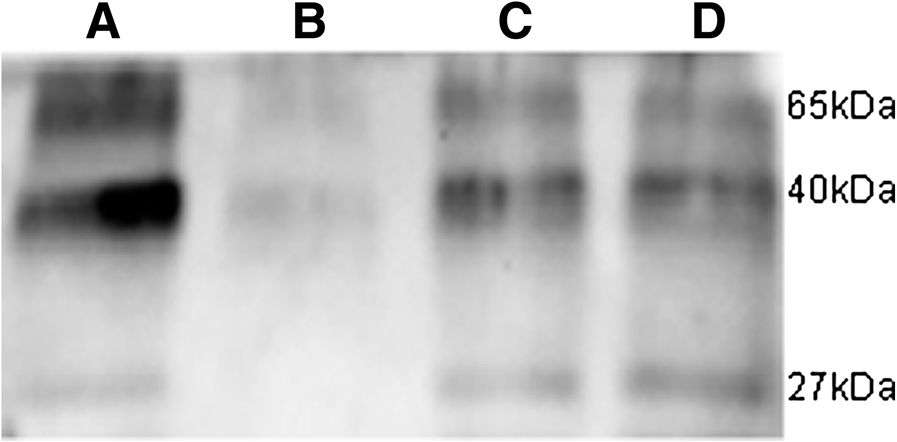
**Western Blot to test PERV-Gag proteins. A**: HEK293 cells infected with PERVs derived from PK-15; **B**: fresh HEK293 cells; **C**: HEK293 cells transfected with WZS-PERV(2); **D**: HEK293cells infected with the virus dirived from the clone.

To confirm the expression of Gag proteins in the infected cells, the total proteins of the cells infected with the virus derived from the clone were extracted and Western blot was employed. As expected, the expression of Gag proteins was observed in HEK293 cells transfected with the clone, the cells infected with the virus derived from the clone and HEK293 cells infected with PERVs derived from PK-15 (Figure 3).

### Indirect immunofluorescence assay

**Figure 4:**
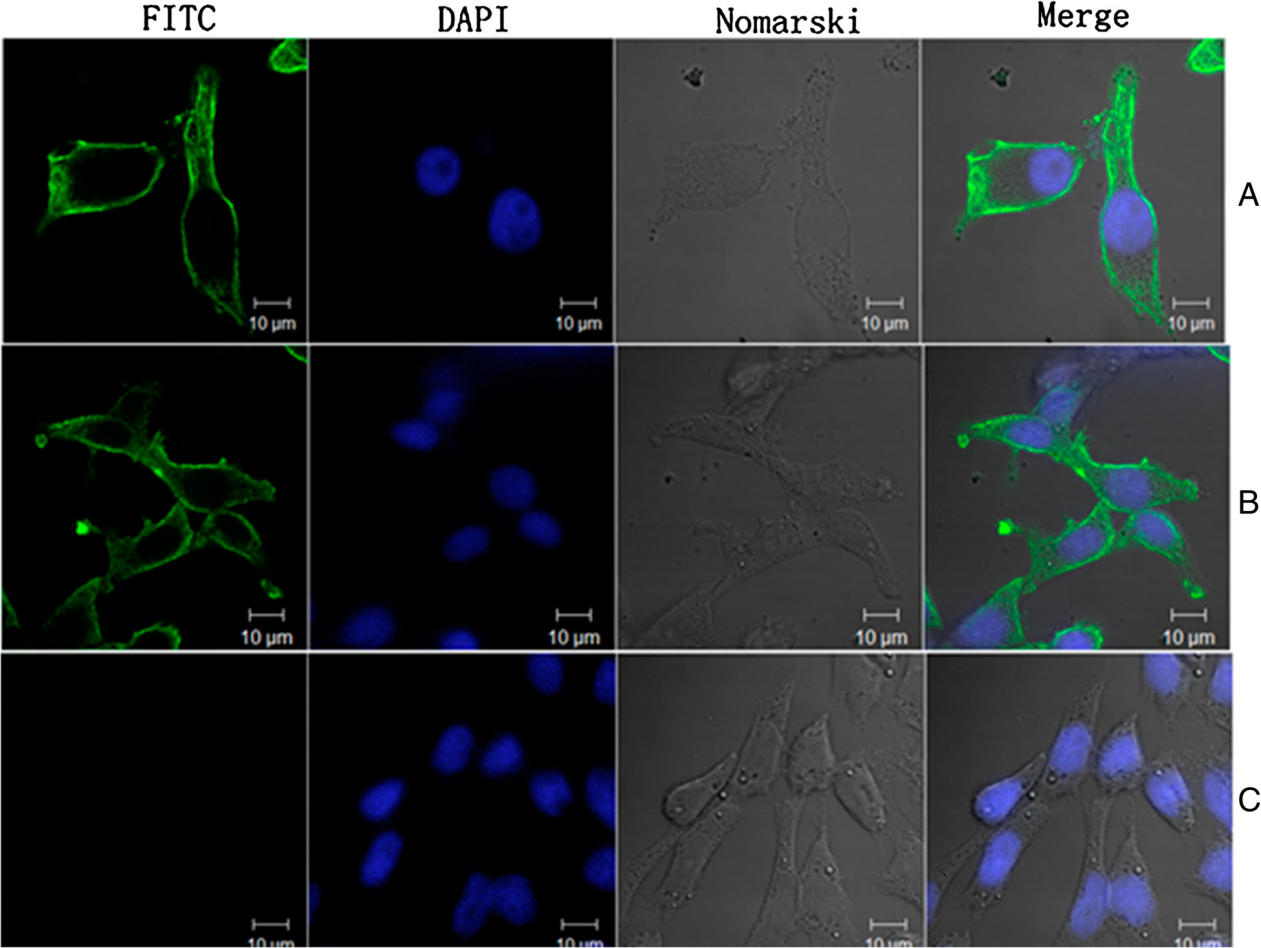
**Detection of PERV Gag expression.** (**A**) indirect Immunofluorescence assay for PK-15cells; (**B**) Immunofluorescence assay for HEK 293 cells infected with the virus derived from the clone; (**C**) Immunofluorescence assay for HEK293 cells as negative. Gag expression was detected in PK-15cells and infected HEK293 cells.

The expression of Gag proteins in the HEK293 cells infected by the virus derived from the clone was investigated by indirect immunofluorescence assay using a mouse anti-PERV-Gag specific antibody. The expression of Gag proteins in PK15 cells and HEK293 cells infected with the virus derived from the clone were observed at 48 hours post-infection, indicating the replication competence of the virus derived from the clone (Figure 4).

### Sequencing and sequence analysis

**Figure 5:**
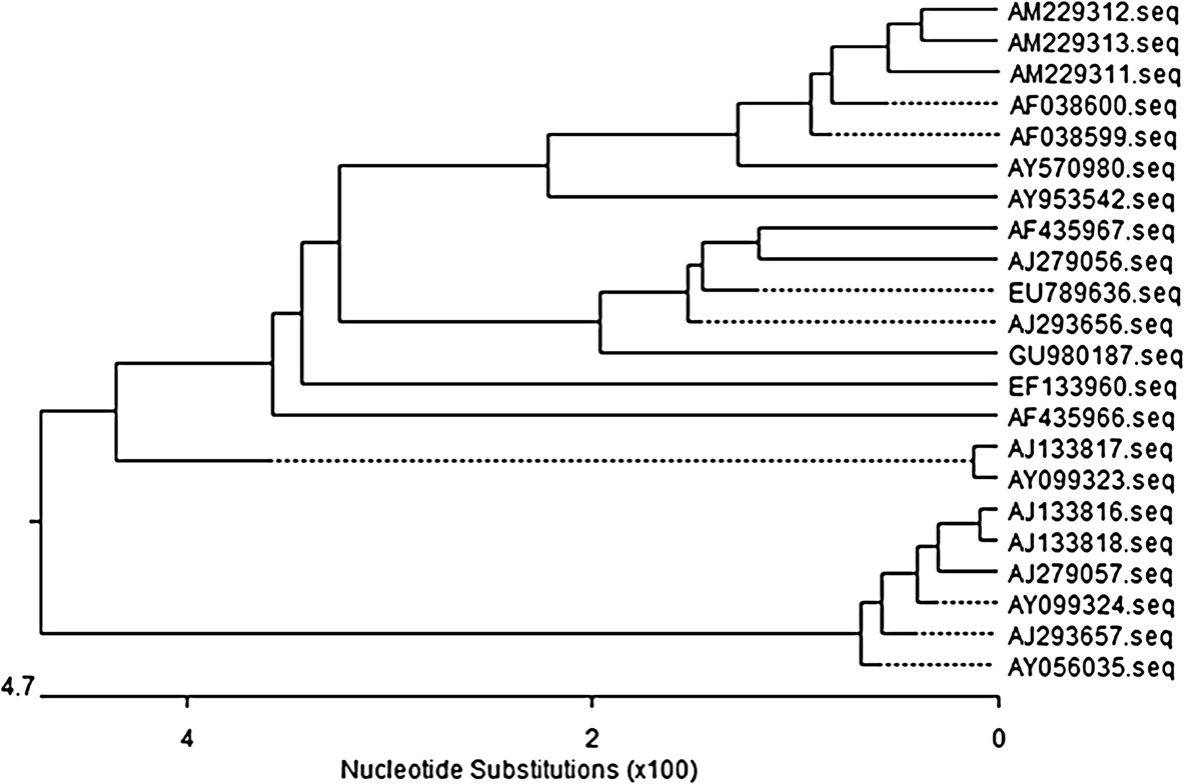
Phylogenetic tree based on the the whole genome of PERVs.

**Figure 6:**
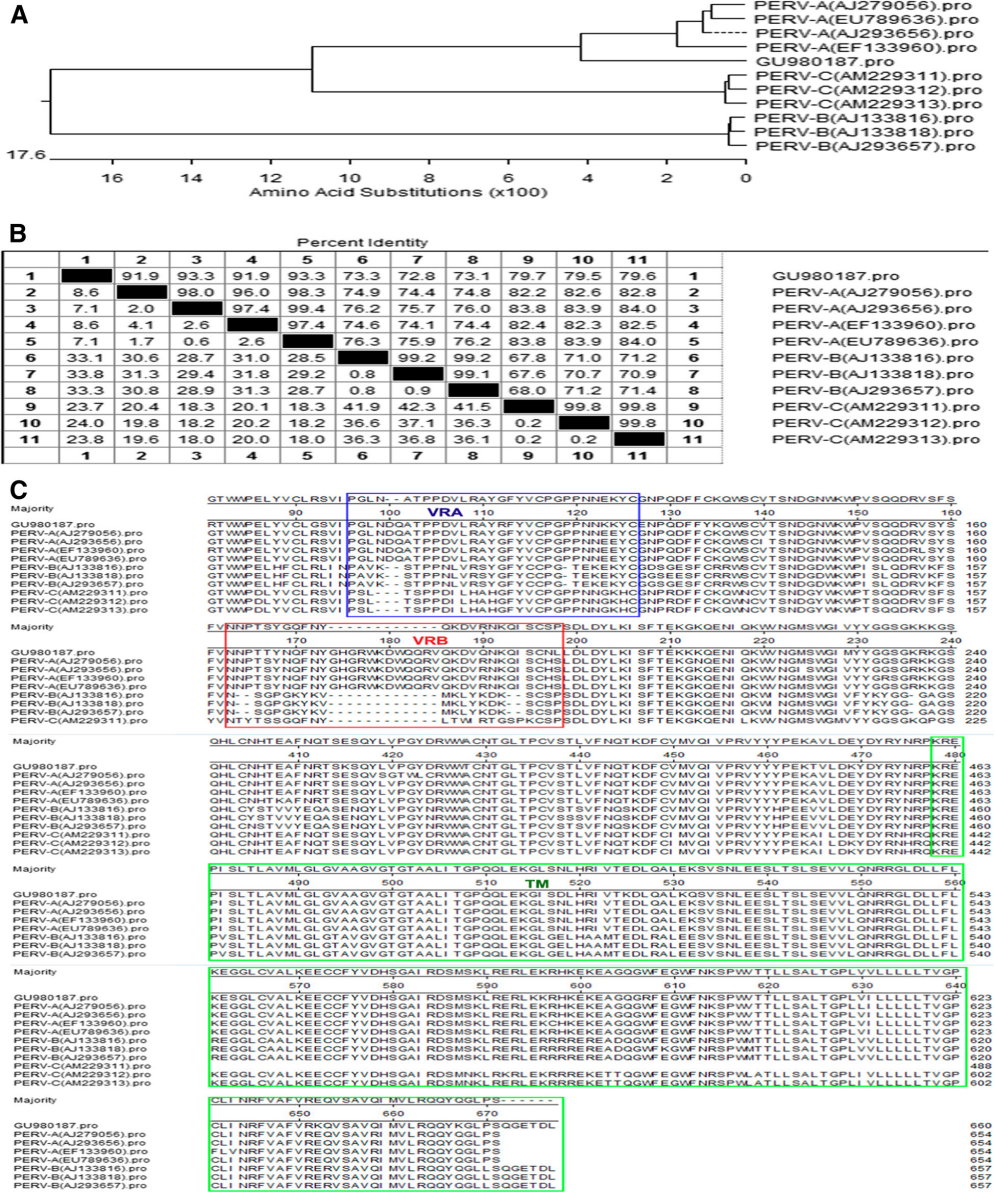
**Comparison of deduced amino acid residues of env of WZS-PERV(2), and PERV-A, PERV-B and PERV-C references.** (**A**) Neighbor-joining trees based on deduced amino acid sequence of envelope glycoproteins of WZS-PERV(2) and PERV-A(GenBank accession No: AJ279056, AJ293656, EU789636 and EF133960), PERV-B (GenBank accession No: AJ133816, AJ133818 and AJ293657) and PERV-C (GenBank accession No: AM229311, AM229312 and AM229313) references. (**B**) Percent Identity of deduced amino acid residues of WZS-PERV(2), PERV-A, PERV-B and PERV-C references. (**C**) Deduced amino acids for the envelope glycoprotein containing variable region A (VRA), variable region B (VRB) and transmembrane (TM) regions of WZS-PERV(2) compared to PERV-A, PERV-B and PERV-C.

**Figure 7:**
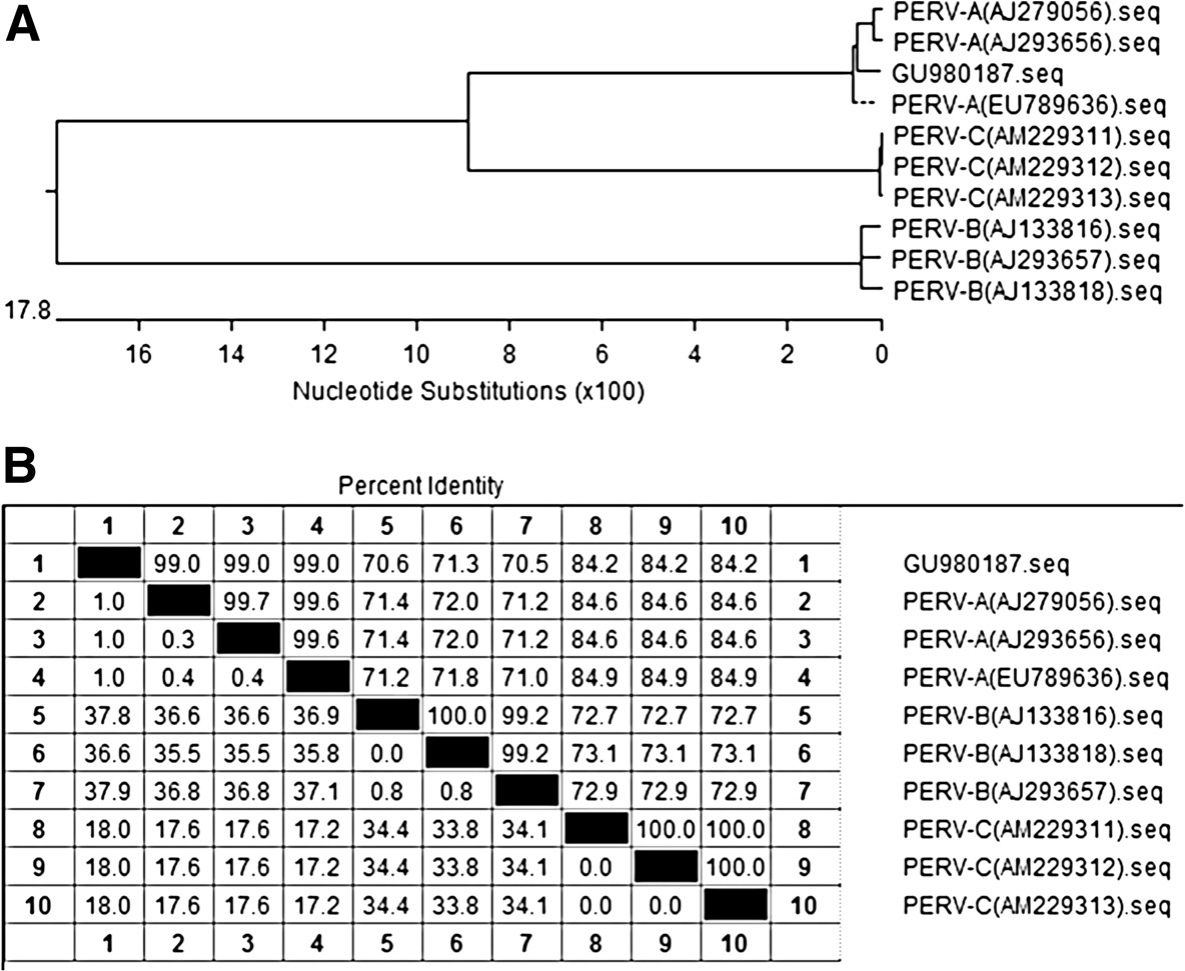
**Comparison of long terminal repeat (LTR) nucleotide sequences of WZS-PERV(2), and PERV-A, PERV-B and PERV-C references.** (**A**) Neighbor-joining trees based on 5’-LTR nucleotide sequences of WZS-PERV(2) and PERV-A(GenBank accession No: AJ279056, AJ293656 and EU789636), PERV-B (GenBank accession No: AJ133816, AJ133818 and AJ293657) and PERV-C (GenBank accession No: AM229311, AM229312 and AM229313) references. (**B**) Percent Identity of deduced amino acid residues of WZS-PERV(2), PERV-A, PERV-B and PERV-C references.

The PERV sequence of WZS-PERV(2) has been deposited in GenBank under accession number GU980187. Sequence analysis showed that the sequence of WZS-PERV(2) had intact *gag, pol,* and *env* open reading frames, as long as 8939 bp. The phylogenetic tree based on the whole genome showed that the nucleotide sequence of WZS-PERV(2) was highly homogenous to two PERV-A references [AJ293656, AJ279056], and was also genetically similar to two PERV-A/C recombinant strains [AY570980, AY953542] and two PERV-C references [AF038599, AF038600], however, had a far relationship with PERV-B references such as AY099324 (Figure 5). To better investigate this viral subtype, local diversity analysis based on the envelope glycoprotein and long terminal repeat (LTR) sequences of WZS-PERV(2) was performed. Alignment of the deduced amino acid sequence showed that the envelope glycoprotein of WZS-PERV(2) was most similar to those of the PERV-A references (data not shown), especially the receptor binding domain containing the variable region A (VRA) and variable region B (VRB), and transmembrane domain (TM) (Figure 6). The LTR nucleotide sequences of WZS-PERV(2) are nearly identical to those of the PERV-A references, with only 1% difference (Figure 7). Similar to other gammaretroviruses is the presence of enhancer like repeat sequences in the U3 region. Interestingly, we found there existed a typical 39 bp (TATTTTAAAAATGA -TTAGTTTGTAAAAGCGCGGGCTTTG) enhancer like repeat sequences in the LTRs region of WZS-PERV(2) with higher similarity to the 37-bp repeat sequences of PERV-C or PERV-A/C recombinant clones with only four base differences [20], but no Pbx1 binding site (TTTGAAATG) was found in this repeat sequences. The Pbx1 binding site is present only in the PERV-C or PERV-A/C repeats [21]. These comparison results combined with the PCR based genotyping result demonstrated that the WZS-PERV(2) belonged to PERV-A subgroup. LTRs of PERV are known to be essential elements in the regulation of retroviral transcription, replication, and integration into the host genomic DNA by various trans-acting factors. Based on the methodology given at the Web site http://mbs.cbrc.jp/research/db/TFSEARCH.html, some important cis-acting elements including AP-1(660–670), CCAAT(237–226), GATA-X(255–266, 328–339), GATA-1(326–338), Evi-1(250–260), MZF1(357–365, 528–536) were identified in the 5’-LTR regions.

Sequence analysis also showed that the *env* gene of WZS-PERV(2) was genetically similar to that of other human-tropic PERV infectious clones. With the *env* gene of PERV-WZSP [GenBank NO: EF133960] as reference, G to A mutant numbers in the env gene of WZS-PERV(2) represented 52.5% of the total mutant base numbers and G to A mutant numbers in the *env* gene of WZS-PERV(2) was amounted to 9.42% of the total G base numbers. APOBEC proteins, as members of a family of cytidine deaminases, have the potential to inhibit the replication of a variety of retroviruses including PERV through a cDNA cytosine deamination mechanism [22-25]. We thus presumed that pA3F might contribute to these mutations, so further studies on the exact mechanism were required.

## Conclusions

In summary, an infectious replication competent clone of PERV from Chinese miniature pigs (WZSPs) designated as WZS-PERV(2) was generated, characterized and sequenced. The replication competence and infectivity in human HEK293 cells *in vitro* were testified by characteristics analysis. The results of sequence analysis and comparison combined with the PCR based genotyping result indicated that the WZS-PERV(2) belonged to PERV-A subtype. In addition, with a previous proviral DNA clone of PERV (PERV-WZSP) as reference, G to A hypermutation occurred in the *env* gene of WZS-PERV(2) was found, whereas APOBEC proteins, as members of a family of cytidine deaminases, have the ability to inhibit the replication of a variety of retroviruses, so these G to A hypermutation might be attributable to porcine APOBEC3F molecule. So further studies on the exact mechanism were required. This infectious clone will contribute to studies on PERV virology and control of PERV in xenotransplantation using Chinese miniature pigs.

## Materials and methods

### Cell culture and preparation of genomic DNA

Human embryonic kidney 293 (HEK293) cells were cultured at 37°C in a humidified 5%-CO_2_ atmosphere in RPMI 1640 medium (Invitrogen) supplemented with 3% fetal bovine serum (FBS, Hyclone), 100 U/ml penicillin and 100 mg/ml streptomycin (Invitrogen). PK15cells were maintained in Dulbecco's modified Eagle Medium (DMEM, Invitrogen) supplemented with 5% fetal bovine serum (FBS, Hyclone).

Blood samples of WZSPs were kindly provided by Institute of Animal Sciences, Chinese Academy of Agricultural Sciences, China. Peripheral blood mononuclear cells (PBMCs) were isolated from these blood samples by Ficoll-Hypaque density gradient centrifugation. The genomic DNA of PBMCs was extracted according to the instructions of Genomic DNA Purification Kit (Promega). The animal experiment was approved by Beijing experimental animal administration committee and performed in accordance with animal ethics guidelines and approved protocols. The animal Ethics Committee approval number is Beijing-SYXK-2008-007.

### PCR-based cloning of the full-length PERV proviral DNA

PCR-based cloning of the full-length PERV proviral DNA was performed as previously described [19]. Briefly, the proviral DNA of PERV was amplified with the genomic DNA of peripheral blood mononuclear cells (PBMCs) from WZSPs as templates in two overlapping halves, that is, 3’ half and 5’ half. The PCR products of PERV-3’ half were cloned into pMD18-T vector (TAKARA) and then digested with Sal I and Xba I, finally inserted into Sal I-Xba I digested pBluescriptαSK^+^ vector. Similarly, the PCR products of PERV-5’ half were firstly inserted into pMD18-T vector, then incorporated into pBluescriptαSK^+^-3’ half using the unique restriction site Nhe I within the overlap between 5’ and 3’ halves. pBluescriptαSK^+^-WZS-PERV recombinant plasmids were thus constructed and identified by colony PCR and restriction enzyme analysis.

### Screening of PERV infectious clones

#### Cell transfection

Altogether 9 clones of pBluescriptαSK^+^-WZS-PERV recombinant plasmids were extracted using Plasmid Midiprep System (Promega),respectively. HEK293 cells were grown to 50%-70% confluence in a six-well tissue culture plate and washed twice with RPMI1640 culture medium. 10 ul Lipofectamine 2000 (Invitrogen) was diluted in 240 ul RPMI1640 (Hyclone) and incubated for 5 min at room temperature. In the parallel, 4 ug plasmids (4 ul of each plasmid at 1 ug/ul) were diluted in 246 ul RPMI1640. These two solutions were mixed gently and incubated for 20 min at room temperature and then the mixture was added directly into the culture medium. The cells were washed three times with RPMI1640 at 6 hours post-transfection and maintained in RPMI1640 supplemented with 10% Fetal Bovine Serium (Hyclone) for an additional 3 days at 37°C in a humidified 5%-CO_2_ incubator.

### RT-PCR and PERV subtype analysis

After transfection for 72 hours at 37°C, the total RNAs of the HEK293 cells trasfected with pBluescriptIISK^+^-WZS-PERV recombinant plasmids were prepared and RT-PCR was performed for *gag*, *pol* and *env* genes to analyze the consequence of cell transfection so that the infectious molecular clones could be selected. According to the classification of *env* gene, PERV subtype analysis was performed based on PCR with three sets of primer pairs, *env-A* (AF: 5’-TGGAAAGATTGGCAACAGCG-3’, AR: 5’AGTGAATGTTAGGCTCAGTGG -3’), *env-B* (BF: 5’-TTC TCC TTT GTC AAT TCC GG-3’, BR: 5’-TAC TTT ATC GGG TCC CAC TG-3’), *env-C* (CF: 5’-CTG ACC TGG ATT AGA ACT GG-3’, CR: 5’-ATG TTA GAG GAT GGT CCT CG-3’) [17].

### Characterization of WZS-PERV(2) clone

#### Transfection of WZS-PERV(2) and infection of PERV

The fresh HEK293 cells was transfected with WZS-PERV(2) recombinant plasmids as described above. The culture supernatant of WZS-PERV(2)-transfected HEK293 cells was harvested, filtered and used to infect fresh HEK293 cells in the presence of 8 ug/ml polybrene.

### PCR and real-time fluorescent quantitative RT-PCR

At 72 hours post-infection, the genomic DNA of the infected cells was extracted using the Genomic DNA Purification Kit (promega) and PCR was performed for *gag*, *pol* and *env* genes to identify the integration between the viruses and the genomic DNA of HEK293 cells. The infected cells were serially passaged, and some infected cells were collected and the genomic DNA was prepared using Genomic DNA Purification Kit (Promega) following the manufacturer’s instructions at every passage time for 51 days. Then PCR was employed with PERV-*pol* specific primer pairs (forward: 5’-TTTCTGGTCATCCCTGAGTGC-3’; reverse: 5’-CCCATCCCTGCGGTTTCT-3’) to detect stability of PERV in the infected HEK293 cells.

In the parallel, to investigate the replication kinetics of PERV virions derived from WZS-PERV(2), the cell-free culture supernatants of the infected cells in triplicate were harvested at every passage time, and the viral RNAs from the supernatants were extracted using Trizol (GIBCO). Then the viral RNAs were reverse transcribed with M-MLV reverse transcriptase (Promega) according to the manufacturer’s instructions, and the number of PERV particles produced from the infected cells was determined with real time PCR using a SYBR qPCR Mix Kit (TOYOBO) as previously described [18].

### Western blot

The total proteins of WZS-PERV(2)-transfected HEK293 cells, the cells infected with the virus derived from the clone were extracted and protein contents were assayed using Bradford method, then subjected to electrophoresis in 12% SDS-PAGE, respectively. The representative spots were cut out and electro-transfered to nitrocellulose membranes, blocked with 5% Bovine Serum Albumin overnight at 4°C. After washed three times with TBS, a 1:1000 dilution of mouse anti-PERV-Gag IgG developed and preserved by our laboratory as the primary antibody were added and incubated for 1 hour at 37°C. After washed three times again, the membranes were incubated with a 1:4000-diluted HRP-conjugated anti-immunoglobulin G secondary antibody. Antibody-antigen complexes were detected by enhanced chemiluminescence (ECL) Kit (Applygen technologies inc.) according to the manufacture’s instructions. Meanwhile, the total proteins of HEK293 cells infected with PERVs derived from PK-15 and fresh HEK293 cells were used as positive and negative controls respectively.

### Indirect immunofluorescence assay

The infected HEK293 cells grown on coverslips were fixed with 4% paraformaldehyde for 5 min at 48 hours post-infection. After washed with 0.01 M PBS and 0.5% Triton X-100 respectively, the infected cells were treated with 1% bovine serum albumin to block non-binding sites. A 1:200 dilution of mouse anti-PERV-gag antibody (Beijing Zhongshan Goldenbridge Biotechnology Company) was added and incubated for 30 min at 37°C, followed by a secondary goat antibody conjugated with FITC solution (Beijing Zhongshan Goldenbridge Biotechnology Company) for 30 min at 37°C and DAPI procedure was employed for 15 min. Finally, coveslips were mounted on slides and examined utilizing Laser Scanning Confocal Microscopy (LSM 510 META). Synchronously, PK15 cells as positive control and HEK293 cells as negative control were setup.

### Sequencing and sequence analysis

The PERV sequence of the WZS-PERV(2) recombinant plasmid was sequenced with the ABI3700 DNA Analyzer (Biolytic Lab Performance Inc.). The WZS-PERV(2) sequence was analyzed with DNAStar software package. Another 21 full-length PERV sequences were retrieved through Blast analysis. Sequence alignments were performed with Clustal W method and phylogenetic tree was conducted using neighbor-joining method.

## Abbreviations

PERV: Porcine endogenous retroviruses; WZSPs: Wuzhishan pigs.

## Competing interests

No competing interests exit in the submission of this manuscript.

## Authors’ contributions

RY, JZ, and YM conceived this study, participated in its design. QY, YM and SX performed the most of experiments and drafted the manuscript. ML, XZ, HY, NZ, JJ participated in partial research work. All authors read and approved the final manuscript.
